# Rapid gene-based SNP and haplotype marker development in non-model eukaryotes using 3'UTR sequencing

**DOI:** 10.1186/1471-2164-13-18

**Published:** 2012-01-12

**Authors:** Tyson Koepke, Scott Schaeffer, Vandhana Krishnan, Derick Jiwan, Artemus Harper, Matthew Whiting, Nnadozie Oraguzie, Amit Dhingra

**Affiliations:** 1 Department of Horticulture, Washington State University, Pullman, WA, USA; 2 Graduate Program in Bioinformatics and Computational Biology, University of Idaho, ID, USA; 3 Horticulture and Landscape Architecture Department, Irrigated Agriculture Research and Extension Center, Washington State University, Prosser, WA, USA

## Abstract

**Background:**

Sweet cherry (*Prunus avium* L.), a non-model crop with narrow genetic diversity, is an important member of sub-family Amygdoloideae within Rosaceae. Compared to other important members like peach and apple, sweet cherry lacks in genetic and genomic information, impeding understanding of important biological processes and development of efficient breeding approaches. Availability of single nucleotide polymorphism (SNP)-based molecular markers can greatly benefit breeding efforts in such non-model species. RNA-seq approaches employing second generation sequencing platforms offer a unique avenue to rapidly identify gene-based SNPs. Additionally, haplotype markers can be rapidly generated from transcript-based SNPs since they have been found to be extremely utile in identification of genetic variants related to health, disease and response to environment as highlighted by the human HapMap project.

**Results:**

RNA-seq was performed on two sweet cherry cultivars, Bing and Rainier using a 3' untranslated region (UTR) sequencing method yielding 43,396 assembled contigs. In order to test our approach of rapid identification of SNPs without any reference genome information, over 25% (10,100) of the contigs were screened for the SNPs. A total of 207 contigs from this set were identified to contain high quality SNPs. A set of 223 primer pairs were designed to amplify SNP containing regions from these contigs and high resolution melting (HRM) analysis was performed with eight important parental sweet cherry cultivars. Six of the parent cultivars were distantly related to Bing and Rainier, the cultivars used for initial SNP discovery. Further, HRM analysis was also performed on 13 seedlings derived from a cross between two of the parents. Our analysis resulted in the identification of 84 (38.7%) primer sets that demonstrated variation among the tested germplasm. Reassembly of the raw 3'UTR sequences using upgraded transcriptome assembly software yielded 34,620 contigs containing 2243 putative SNPs in 887 contigs after stringent filtering. Contigs with multiple SNPs were visually parsed to identify 685 putative haplotypes at 335 loci in 301 contigs.

**Conclusions:**

This approach, which leverages the advantages of RNA-seq approaches, enabled rapid generation of gene-linked SNP and haplotype markers. The general approach presented in this study can be easily applied to other non-model eukaryotes irrespective of the ploidy level to identify gene-linked polymorphisms that are expected to facilitate efficient Gene Assisted Breeding (GAB), genotyping and population genetics studies. The identified SNP haplotypes reveal some of the allelic differences in the two sweet cherry cultivars analyzed. The identification of these SNP and haplotype markers is expected to significantly improve the genomic resources for sweet cherry and facilitate efficient GAB in this non-model crop.

## Background

Sweet cherry (*Prunus avium* L.), a non-model crop, is an important non-climacteric member of sub family Amygdoloideae where other members like peach and plum demonstrate climacteric fruit ripening. Sweet cherry is a diploid (2n = 16) and is estimated to be slightly larger than peach, 225-300 MB [1,2]. Sweet cherry underwent a recent breeding-related genetic bottleneck that reduced the diversity present in the germplasm [3]. Genetic variability can be utilized to screen for resistance to diseases and improve the efficiency of selecting desirable genotypes through breeding especially in sweet cherry where natural diversity is lacking. Types of variation at the nucleotide level are: microsatellites or simple sequence repeats (SSRs), single nucleotide polymorphisms (SNPs), insertions and deletions (indels) and genomic rearrangements [4]. Identification of genetic diversity in species which lack significant genomic resources has typically been a time-consuming and laborious process.

SSR markers have been used extensively for population genetics and genome mapping studies in several members of Rosaceae [5,6]. SSR identification techniques are typically costly and time consuming [7-9]. Most published SSRs are located in the intergenic regions [4]. A recent study in *Populus* attempted to identify SSRs in exons or expressed gene fragments. The abundance of microsatellites within the coding region was three-fold lower than intergenic regions and, when present, microsatellites do not show useful allelic variability. Further, the authors concluded that candidate gene approach for development of microsatellites may not be the best strategy [4]. While SSRs remain difficult to develop, SNP identification and validation has rapidly improved in past years mostly due to reduction of sequencing costs. Previously, direct sequencing of a gene of interest related to supernodulation was used to identify SNPs [10]. Similar studies in non-model species lacking such resources require sequence information from related species. SNPs have also been used for anchoring a linkage map and bovine genome [11]. Ganal et al. [12] reviewed recent SNP identification methods including DNA arrays, amplicon sequencing, mining existing EST resources, and using sequence data generated with second generation sequencing technologies. Compared to other methods, re-sequencing applications were determined to produce a higher percentage of validated SNPs, while non-reference based next-generation sequencing, or *de novo*, approaches required the least amount of *a priori* genetic/genomic information. A major caveat of using second generation sequencing *de novo* is the ability to acquire sufficient depth to accurately identify SNPs. Therefore, a reduced representation sequencing approach was recommended. Many reduced representation methods integrating high throughput sequencing are discussed by Davey et al. [13] and the authors further elaborated on the utility of SNP-based molecular markers.

Continued improvements in second generation DNA sequencing technologies have increased the ability to obtain significant sequencing depth in a rapid and cost efficient manner, compared to Sanger sequencing approaches [14]. Bundock et al., [15] performed amplicon sequencing on genes of interest with 454 technology to produce a large number of reliable SNPs from two parents of a QTL mapping population of sugar cane finding high success rates for SNP verification (93%). Recently, next generation technologies have been widely utilized for sequencing transcriptomes of various species [16-18]. Eveland et al. [19] reported a quantitative transcriptomics approach based on selective sequencing of the 3'UTR of mRNA from *Zea mays*. Their work demonstrated a clear ability to resolve the expression of nearly identical genes (99% nucleotide identity) based on variation in the 3'UTR (97% nucleotide identity). Through comparison with sequences in multiple maize databases, 93.8% of the SNPs identified by Eveland et al. were confirmed [19]. Use of a 3'UTR directed approach exploits the higher number of variations found in the 3'UTR region compared to the coding region of a gene. Higher sequence variation, combined with physical linkage to a specific gene, increases the potential impact of 3'UTR polymorphisms in connecting genetics and functional genomics studies especially in non-model eukaryotes. This is in contrast to current approaches where intergenic polymorphisms are used for scoring a segregating phenotype without the associated gene-related information. The method presented here utilized the positive aspects of 3'UTR sequencing, as a reduced representation approach, to facilitate rapid gene-linked SNP identification.

In addition to identifying polymorphisms, current research in human genomics has demonstrated the utility of developing haplotype information as a way to more fully understand genotype to phenotype relationships, especially in context of health, disease and response to environmental cues [20-22]. Generally, haplotypes are comprised of allelic variants on each of the two chromosomes at the same locus, though the definition and utilization varies in application from linking multiple polymorphisms across several loci down to multiple polymorphisms in a single gene [23]. Additionally, haplotype determination has been aided by DNA strand specific or genomic phase-based information generated using second generation sequencing technologies since each sequencing read is from only one homologous chromosome and not a consensus of the two [24]. Similarly, next generation RNA-seq and 3'UTR sequencing has the ability to reveal haplotypes within a gene [25] and thus enable identification of allele specific sequence and its expression simultaneously. Here we present our approach that utilizes 3'UTR sequencing to rapidly develop SNP and haplotype markers in sweet cherry, a species without a published genome sequence and a non-model crop. Through *de novo* assembly of 454 generated-3'UTR sequencing reads and strict filtering, we initially identified a putative set of contigs containing SNPs. Primer sets designed to amplify the regions of these contigs with putative SNPs were developed and used for High Resolution Melting (HRM) analysis among eight currently utilized parental cultivars of sweet cherry and 13 hybrid seedlings derived from a cross between two of the parental cultivars, respectively. We determined that 68 out of 223 (30.5%) and 65 out of 217 (30.0%) of the tested primer pairs are able to detect genetic variability. From these polymorphic sites, 685 haplotypes were identified from 301 contigs containing multiple SNPs.

## Methods

### RNA Extraction and cDNA preparation

Tissue samples from developing floral buds of two commercially important cherry cultivars, Bing and Rainier, were excised from the trees and flash frozen in liquid nitrogen. The frozen tissues were pulverized uniformly in a SPEX SamplePrep 6870 FreezerMill (SPEX SamplePrep, Metuchen, NJ) for five cycles each with cooling for two minutes and grinding at 15 counts per second for four minutes. Total RNA from each sample was extracted using the RNeasy Plant DNA Extraction Kit (Qiagen, Germany). First strand cDNA was then synthesized using the Ambion aRNA synthesis kit with a biotinylated poly-T B-adaptor [see Additional File 1 for adaptor sequences] for 3'UTR profiling as described by Eveland et al. (2008). Second strand cDNA was created, cleaved with MspI, and ligated to modified A-adaptors containing indexing tags [see Additional File 1 for adaptor sequences] as per the Eveland protocol.

### Sequencing and assembly

The 3'UTR libraries were sequenced as per the 454 FLX protocol (Roche, USA) on a single LR-70 sequencing plate. After sequencing, the 454 produced reads were processed using a custom script [see Additional File 2] to remove the multiplexing barcode and rename each read with its appropriate sample name at the end of the header. All of the modified reads were then assembled using SeqMan from the Lasergene 7 suite (DNASTAR, Madison, WI).

### SNP Identification

For method development, a total of 10,100 contigs were examined for the presence of putative SNPs using Lasergene 7's SeqMan (DNASTAR, Madison, WI). The high confidence SNPs have at least two alleles represented by a minimum depth of three reads per nucleotide call per allele. Primer pairs flanking potential SNP loci were designed using the PRIMER3 program [26] to amplify 50-100 base pair amplicons. This yielded 223 primers from regions of 207 contigs for HRM analyses.

### Population Variation Screen

Eight sweet cherry cultivars: Bing, Chelan, Emperor Francis, New York 54, Regina, Selah, Stella and Cowiche used as parental material in the Washington State University (WSU) Sweet Cherry Breeding Program (Prosser, WA) were used to test the polymorphisms of the identified SNP loci across Bing and Rainier cultivars. For segregant analysis, 13 seedlings from an F_1_ mapping population of Selah × Cowiche were used. Leaves of these accessions were collected from the WSU Irrigated Agriculture Research & Extension Center in Prosser, WA and DNA was extracted from dried leaves using a CTAB extraction protocol [27]. The reaction mixture for HRM analysis consisted of 0.6 μL of each primer (10 μM), 12.0 μL SYBR^®^ Green, 5 ng of genomic DNA and autoclaved nanopure water to a total volume of 20 μL. The Cultivar panel comprised of 223 primer sets tested on all eight parental cultivars and the Seedling panel included 217 primers sets tested on one reaction of each parent, Cowiche and Selah, and one of each hybrid seedling. Analyses were performed on the LightCycler^®^ 480 System (Roche Branford, CT) using the following PCR cycling and HRM conditions. Initial melting for 10 minutes at 95°C was followed by 45 cycles of 95°C for 10 seconds, 57°C for 15 seconds, and 72°C for 15 seconds, then heated to 95°C for 1 minute and cooled to 40°C. High Resolution Melting analysis was then automatically initiated whereby the amplicons were heated from 60°C to 90°C with 25 acquisitions per degree. As the temperature slowly increased, the dye fluorescence was recorded, plotted and later analyzed using the LightCycler^®^ 480 Gene Scanning Software. Since the T_m_ can vary based on the HRM reaction conditions, curve shapes were visually examined and the number of distinct curve profiles was identified for each primer set.

### Secondary Assembly and SNP reporting

After the HRM analysis, a second assembly using SeqMan NGen v3.0 (DNASTAR, Madison, WI) was performed due to its improved algorithm and the results were used for SNP reporting on the entire data set. This assembly was completed using the default parameters for NGen 3.0's *de novo* transcriptome assembly of: 85% match, match size 21, genome length 225 MB. The whole SNP report was initially filtered to retain the HRM confirmed SNPs using a minimum total depth of 10 reads at the polymorphic base and at least 20% variance from the consensus. Further filtering into high confidence SNPs was performed by screening for at least two alleles represented by a minimum depth of three reads per nucleotide call per allele. This minimum depth per allele for each SNP equals or exceeds the published depths using either 454 data [28,29] or Illumina data [30,31]. Additionally, SNPs resulting from the first or last five bases of reads were rejected. The transition and transversions ratio (R value) was determined by summing all of the transitions (C/T and A/G) and transversions (A/C, A/T, C/G, and G/T).

### Haplotype Identification

Haplotypes were identified visually by analyzing the combined transcriptome assembly generated using NGen 3.0 in SeqMan (DNASTAR). Similar to the SNP screening, at least three reads of an allele spanning two SNP loci were required to link SNPs into a haplotype. When two or more haplotypes were present at one locus, they were differentiated and recorded as separate haplotypes for their use as haplotype markers.

## Results and Discussion

### Method Overview

**Figure 1:**
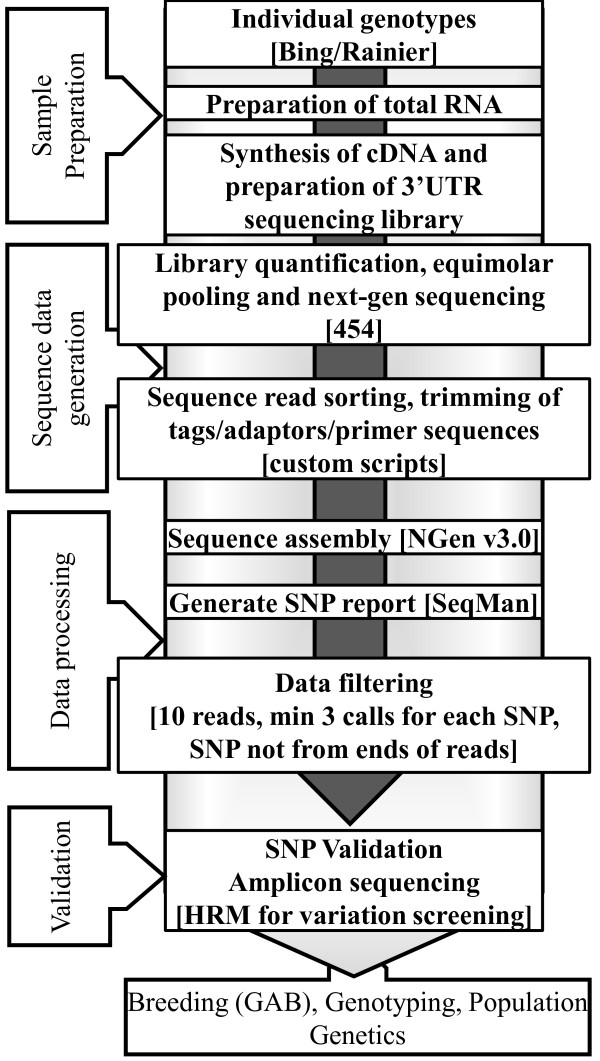
**General schema for rapid identification of SNPs**. The method consists of four stages 1. Sample preparation, 2. Sequence data generation, 3. Data processing and 4. Variation screening or validation of polymorphism. Content in parentheses denotes the materials, software and methods used in this study. The variable polymorphic regions can facilitate efficient gene assisted breeding (GAB), genotyping and population genetics studies.

The general method presented in this study is based on four steps as outlined in Figure 1. The first step of sample preparation involves identification of appropriate individuals across whom genetic polymorphism needs to be determined. In our study, we used two closely related sweet cherry cultivars to test our approach. However, it is recommended that phenotypically diverse individuals should be chosen. Additionally, the number of individuals can be increased as desired keeping in mind the expected transcriptome size and the number of sequencing reads expected to be generated by the next generation sequencing platform that will be employed for transcriptome sequencing in step 2. This parameter is critical for strict filtering of data for identification of SNPs in step 3. Total RNA needs to be extracted from tissues which are representative of the phenotypic diversity between the samples. Developing reproductive buds used in this study were derived from Bing and Rainier each grafted onto two rootstocks Mazzard and Gisela 6. Bing and Rainier grafted on Gisela 6 yielded fruit that was 656% to 212% more than the same cultivars grafted on Mazzard [32]. The RNA is converted into cDNA and further processed for selection of 3'UTRs [19]. In step 2, after extensive quantification of the 3'UTR libraries, samples are pooled in equimolar ratios and sequenced using next-generation sequencing platforms. At the time, we performed pyrosequencing on the 454 GS FLX instrument since it provided the longest read lengths. However, at present, such a method would benefit greatly from Illumina or SOLiD platforms since the read lengths have greatly improved [33]. Depending on the sequencing platform the raw sequence data needs to be pre-processed by trimming of tags and adaptor sequences prior to moving to step 3 of data processing where the sequence data is assembled. We used the NGen v3.0 (DNASTAR, Madison, WI) assembler and the output was visualized using SeqMan which generated a SNP report. The final set of SNPs was selected using strict parameters as outlined in the materials and methods. In step 4, putative SNPs were tested for variability across 8 parental cultivars and 13 progeny derived from a cross between two cultivars using HRM analysis. Utilization of SNPs for screening variability in population has been well documented in literature [34-36]. Subsequently, for SNP validation, barcoded amplicon sequencing for a very large number of markers (SNP or haplotype) across a large array of progeny in a segregating population or genetic collection would be an efficient approach. For smaller number of samples or for initial confirmation of variation, techniques such as HRM may be more appropriate as utilized in this case. Rapid identification of gene-linked polymorphisms as proposed in this method can facilitate efficient Gene Assisted Breeding (GAB), genotyping and population genetics studies in non-model eukaryotes.

### Sequencing and Assembly of 3'UTRs

Pyrosequencing of 3'UTR libraries from Bing and Rainier on a single 454 GS FLX sequencing plate produced a total of 580,455 reads (Table 1). The reads had an average length of 85 bp which is as expected from the 454 GS FLX sequencing platform and the 3'UTR library preparation. The reads were processed with a custom script to trim index sequences and label the headers appropriately [see Additional File 2]. Transcriptome assembly of the trimmed sequences with SeqMan 7.0 (Lasergene Suite 7.0, 2009) yielded 43,380 contigs.

**Table 1:** Summary of 3'UTR sequencing results

Sample	Bing	Rainier	Total
Number of bases	25409323	23893350	49302673

Number of reads	303684	276771	580455

Avg. read length	83	86	85

The table represents number of bases, reads and average read lengths generated for Bing and Rainier cultivars.

### Initial SNP identification

To test our experimental approach, analysis of a subset of the assembled contigs was performed to identify SNPs within the dataset. The 100 contigs with the highest number of reads and contigs 1-10,000 as produced by SeqMan 7 (Lasergene Suite 7.0, 2009) were analyzed yielding 600 contigs containing at least one high confidence SNP. These high confidence SNPs have at least two alleles represented by a minimum depth of three reads per nucleotide call per allele. Since false polymorphism of indels can be high [37], indels were not included in this analysis to avoid identification of false polymorphisms as previously described [38]. The total number of SNPs in this dataset was not calculated as only the described subset was examined. A total of 223 primer sets were designed from 207 contigs with PRIMER3 [26] to amplify the small regions around the identified SNPs [see Additional File 3 for primer sequences and associated contigs].

### Population Variation Screening

**Figure 2:**
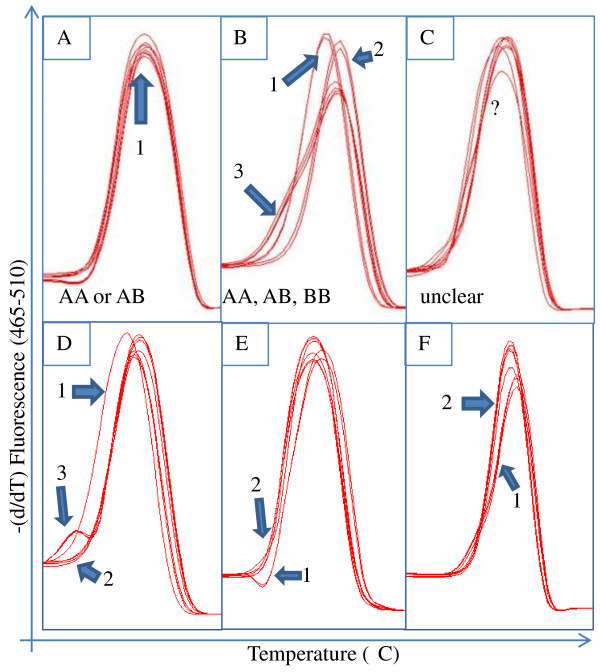
**Analysis of variation of identified SNPs via high resolution melting (HRM) curves generated on 8 cultivars used in this study**. HRM derivative plots, -(d/dT) fluorescence as a function of temperature, of several primer sets when analyzing 8 sweet cherry cultivars representing the common patterns observed during analysis. Comparisons outside one frame are not meaningful and the frames are not to scale with each other as the curve shape is the focus. **A-C** are from primers amplifying a region expected to contain 1 putative SNP while **D** contains 2, **E** has 3, and **F** contains 5. **A**. Primer set 121 produces a single curve pattern denoted by an arrow representing either a homozygous locus across all 8 cultivars tested or a heterozygous locus shared by all 8 tested cultivars. **B**. Primer set 100 has three distinct curve patterns highlighted as 1, 2 and 3 representing three allelic forms at the sampled locus. **C**. Primer set 115 has an indiscernible pattern. **D-F**. Each demonstrates variation in the population; however, the more SNPs present in the amplified region the smaller the differences among the melt curves.

**Figure 3:**
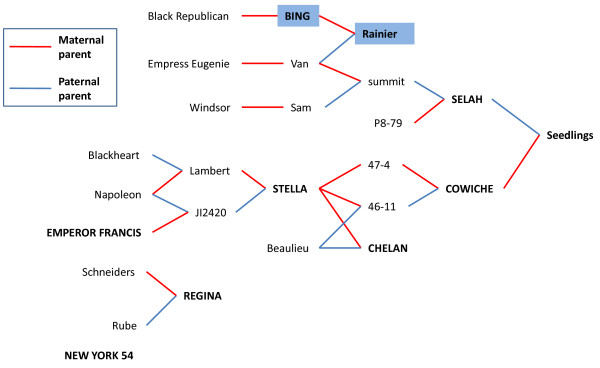
**Pedigree relationships of the 8 cultivars used in this study**. Pedigree of the sweet cherry cultivars used for SNP development (blue box) and those used for HRM analysis of the SNPs (bold and all caps). The maternal parent is marked by a red line and the paternal parent by a blue line.

**Figure 4:**
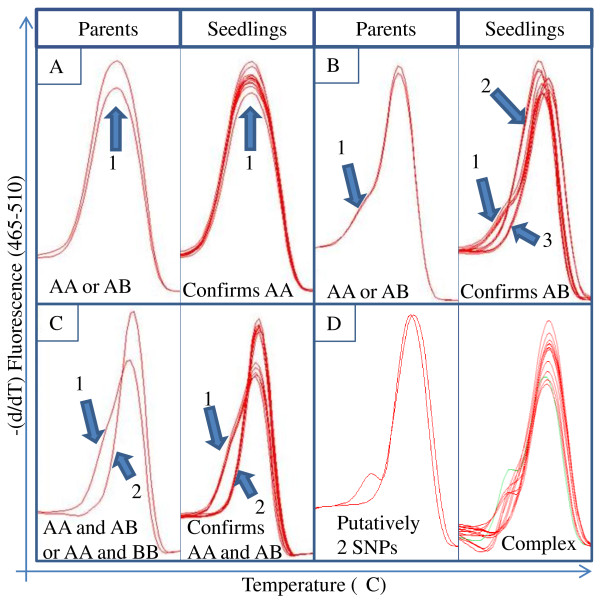
**Four primer sets with the HRM curve for the two parents, Cowiche and Selah, on the left and the 13 seedlings on the right**. HRM derivative plots, (-d/dT) fluorescence as a function of temperature. **A-C** contain 1 putative SNP while **D**. contains 2 putative SNPs. **A**. Primer set 131 shows no variation as expected for crossing two of the same homozygotes. Note the single curve profile in both the parents and seedlings. **B**. Primer set 189 shows a single curve profile in the parent panel which differentiates into a 1:2:1 (3:6:4) genotype ratio as expected for a heterozygous × heterozygous cross in the seedlings. It is represented by three different curve profiles where profile number 1 corresponds to the heterozygous parental profiles. **C**. Primer set 100 shows a 1:1 (8:5) genotype ratio as expected in a homozygous and heterozygous cross. The curve profiles are similar between the seedlings and the parents. **D**. Primer 92 shows 2 parental curve types. However, the seedlings show several distinct curve types which is not unexpected due to the presence of 2 high quality SNPs in this region.

The automated genotype calling of the LightCycler 480 analysis software v1.5.0 identified only a few SNPs with more than one allele. Modifying the analysis parameters did not provide significant improvement of the automated analysis (data not shown). However, manual analysis identified multiple curve types for many primer sets as well as heritable patterns between Cowiche, Selah, and their seedlings. While the differences in melting curve shape are small, homozygotes and heterozygotes were visibly distinguishable with many of the primer sets (Figures 2A &2B). It is unclear why the HRM curves presented in this manuscript differ from those shown by Wu et al. [39]. These smaller changes in the derivative plots could be due to the larger amplicon size (~150 bp). Manual analysis of the Cultivar and Seedling tests indicated that 68 out of 223 (30.5%) and 65 out of 217 (30.0%), respectively, of the designed primer pairs displayed variation with 49 pairs showing variation in both tests (Table 2). This is expected as it is recommended to design three primer sets for each SNP of interest according to ABI's guide to HRM [40] analysis which suggests that a success rate of 33% is typical. A total of 23 primer sets from the Cultivar panel and 19 from the Seedling panel, were considered non-variant for this experiment since they displayed indiscernible variation (Figure 2C). Additionally, it became evident during the analysis that multiple SNPs in an amplified region made distinction more difficult, though it was still possible in the best cases (Figure 2D-F). Eight of the non-variant primer sets were shared between the two panels. Reactions which did not produce a curve in either panel were labeled 'failed'. Some of the failed primer sets produced amplicons on one of the two panels suggesting amplification issues. Ten primer sets failed in both panels, most likely due to an error either in the contig sequences or the primer design. Overall, 84 of the 217 (38.7%) primer sets used on both panels showed variation in one or both sets. The remaining 61.3% of the SNPs did not have detectable variation in the individuals tested. One explanation for this is that the tested cultivars mathematically only represent 12.5% of the alleles from Rainier's paternal parent, Van, based on the pedigrees of the tested cultivars (Figure 3). Alternatively, lack of detection may be a result of the amplicon length hindering the ability to visualize the melting differences between variants. This variation detected by HRM was far lower than the detection from amplicon sequencing of sugar cane though the sugarcane work focused on genes of interest whereas we used a *de novo* approach [15]. The authors had screened for SNPs in polyploid parents and the resulting progeny. It is critical to note that in this work, we identified SNPs from two cultivars and then validated them across 8 parental cultivars, 6 of which are not closely related. Additionally, the progeny used for SNP variation screening are far removed from the genotypes used for initial SNP discovery. Most importantly, sweet cherry has a narrow genetic diversity further reducing the possibility of identifying a large number of SNPs. Our work clearly illustrates that sequencing and assembly based method for identification of SNPs is highly effective and that the HRM screen is likely a limiting step. Heritability of the curve types can also reveal cultivars that are homozygous at a given locus (AA × AA) or heterozygous (AB × AB) (Figure 4A &4B). Additionally, the Seedling HRM curves can confirm that one parent is homozygous and the other heterozygous with an approximately 1:1 ratio (8:5) of curve types matching the two parents (Figure 4C). Though higher numbers of individuals need to be tested to obtain statistical significance, noting that these patterns are distinguishable through HRM provides a foundation for the use of this method to screen progeny or parents to determine their allelic composition.

**Table 2:** Experimental assessment of SNPs

		Cultivar panel		Seedling panel (Selah × Cowiche)		
**Type**	**Number tested**	**Number with variation**	**Number failed**	**Number tested**	**Number with variation**	**Number failed**

**SNP primer sets**	223	68	15	217	65	11

A total of 223 predicted SNP sites were tested via HRM in 8 cultivars and 217 predicted SNP sites were tested in 13 seedlings derived from two of the cultivars. The table represents the results of these HRM analyses.

### Secondary Assembly and SNP reporting

DNA assembly programs continued to improve since the initial assembly which was used to design primers and analysis of population variability. Subsequently, the trimmed reads were re-assembled using NGen v3.0 (DNASTAR, 2011). This assembly produced 34,620 contigs [see Additional File 4] with an average length of 149 bp (Table 3). Since the aim was to obtain high depth of coverage of around 100 bp upstream of the poly-A tail, the longer contigs were unexpected. Analysis of this issue confirmed that the poly-T sequence containing primer used for first strand cDNA synthesis annealed to some poly-A regions in coding regions of the transcripts as well. While not all the sequencing reads were from the direct vicinity of the poly-A tail, the contigs remain gene-linked due to their cDNA origin. This could reduce the total number of identified SNPs since genic regions have a greater selection against mutations when compared to the 3'UTR as previously described [19].

After filtering the new SNP report for a minimum read depth of 10 and 20% variance from the consensus an initial list of SNPs was derived. These SNPs were examined to remove any SNPs resulting from the ends of the reads and filtered requiring a minimum of three confirming reads per base call per allele. A total of 2243 putative SNPs were identified in 887 contigs after this filtering [see Additional File 5]. These data, consisting of contigs and SNPs, have been uploaded to NCBI (GenBank JP376615-JP382830 and dbSNP NCBI ss# 469992783-469995036 except 469992784, 469992792, 469992801, 469992809, 469992818, 469992823, 469992825-7, 469992834-5, 469992842, 469992851, 469992853-4, 469992859, 469992866-7). Analysis of the "failed" HRM primer sets on contigs obtained from the NGen assembly showed that none had a significant change in the contig consensus. However, three of the 10 primers did show multiple possibilities for primer binding which could decrease PCR effectiveness.

**Table 3:** Transcriptome assembly results

Assembly Version	NGen v3.0
Number of contigs	34,620

Avg. contig length	149

Median contig length	118

Total contig bases	5,191,475

Number of putative SNPs	2,243

Statistics on the transcriptome assembly for the sequence data generated for Bing and Rainier developing floral buds.

The 2243 putative SNPs identified in the assembled gene space (expressed sequences) of 5.19 Mb yields a SNP frequency of 1 in 2,315 bp (0.43 SNPs per kb of gene space). The sweet cherry gene space of 5.19 MB generated in this study represents approximately 2.3 - 1.7% of the estimated genome size of 225 - 300 MB. Previous studies utilizing whole genome sequence have reported a frequency of 1 SNP in 114 bp (8.8 SNPs per kb) and 1 SNP in 208 bp (4.8 SNPs per kb) in almond *Prunus armenica* (genome size = ~200 MB) and apple *Malus* × *domestica* (genome size = 740 MB) respectively [39,41]. The recent genetic bottleneck and Bing being a parent of Rainier reduces the number of potential alleles present in the dataset to 3 whereas the almond and apple studies examined 25 and 5 cultivars respectively.

As mentioned earlier, coding regions of genes were also sequenced inadvertently since the poly T primer annealed to regions other than the 3'UTR region, thereby further reducing the number of polymorphic sites in the sequenced regions. Analysis of the putative sweet cherry SNPs for transitions (C/T and A/G) and transversions (A/C, A/T, C/G, and G/T) yields a transition to transversion ratio (R value) of 1.14/1 (Table 4). This is nearly identical to the 1.16:1 ratio found across 25 almond cultivars [39] and differs slightly from the 1.27:1 ratio in *Prunus mume*, Japanese apricot [42].

**Table 4:** Summary of transitions and transversions across Bing and Rainier

Transitions	
C/T	598

G/A	595

Total	1193

**Transversions**	

A/C	242

A/T	348

C/G	158

G/T	298

Total	1046

Each SNP was classified based on the base change that occurred. The total number of transitions (a sum of the C/T SNPs and A/G SNPs) is 1193 which is marginally greater than total number of transversions (A/C + A/T + C/G + G/T) which is 1046. The R value (transitions/transversions) is 1.14 as expected within a species.

### Haplotype Identification

**Figure 5:**
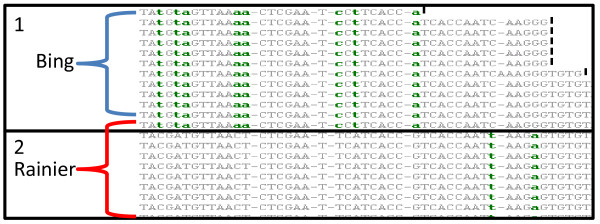
**Screenshot of the SeqMan (DNASTAR) visualization of contig456 showing 2 alleles at a single locus**. Boxes 1 and 2 represent unique haplotypes obtained from the NGen 3.0 assembly of the 454 reads from Bing and Rainier according to the filtering parameters described in the methods. These haplotypes differ at each of the bases labeled in green on one of the haplotypes for a total of 10 SNPs between these haplotypes. Haplotype 1 consists of 11 reads from Bing and 1 of Rainier while haplotype 2 is entirely Rainier.

From the final SNP report, contigs possessing more than one high quality SNP were analyzed for the presence of haplotypes. The sequence and base position for each distinguishable haplotype of the contig were detailed [see Additional File 6]. An example of a haplotype containing 10 SNPs at a single locus (Figure 5) demonstrates clear differences between the two haplotypes. In total, 301 contigs contained at least two haplotypes and 15 had more than two haplotypes in a region. Additionally, 34 contigs had multiple, unlinked haplotype regions that likely represent sections of haplotypes that, due to lack of read length or inadequate depth of sequence reads and the previously stated requirements, were not able to be linked in this analysis. In total, this amounts to 685 unique haplotypes over 335 loci in 301 contigs. Development of these haplotype blocks is expected to greatly benefit sweet cherry breeding efforts specifically, but warrant consideration for future phylogenetic and comparative genomic studies in other related species as well. As haplotypes, these SNP blocks also represent loci that may be extremely useful for development of molecular markers like CAPS. Since these haplotyped SNPs are inherited as a block, future studies would benefit from a higher depth of coverage to ensure complete linkage of haplotype blocks. It is acknowledged that the linked SNPs are very close in the short contigs, and they tend to be haplotypes due to low probability of recombination between them. However, such haplotypes are highly relevant to the current short read sequencing platforms where shorter reads of 50 to 100 bp can be utilized to accurately identify an allele in a diploid or polyploid sample or detect mutations that may occur individually creating a new haplotype.

### Access to Sequence and SNP data

Due to the nature of the contigs and SNPs, many of them did not fit the requirements for typical submission to NCBI. All contigs and high quality SNPs are available as additional files to this manuscript. All of the raw sff files were uploaded to NCBI's Sequence Read Archive (SRA046001.1). Contigs greater than or equal to 200 bp in length were added to GenBank's Transcriptome Sequence Assemblies (TSA) database (GenBank JP376615-JP382830) as *Prunus avium* assemblies and SNPs corresponding to these sequences larger than 200 bp were uploaded to dbSNP (NCBI ss# 469992783-469995036 except 469992784, 469992792, 469992801, 469992809, 469992818, 469992823, 469992825-7, 469992834-5, 469992842, 469992851, 469992853-4, 469992859, and 469992866-7).

## Conclusions

A method for developing gene-linked SNP and haplotype markers through high-throughput 3'UTR sequencing for species lacking genome sequences was demonstrated. Through this process, 2243 putative SNPs were identified and 34,620 contig sequences were obtained and added to NCBI database for use by the plant research community. To our knowledge, the 685 haplotypes developed in this study are the largest set of reported SNP-based haplotypes in sweet cherry and demonstrates that haplotypes can be identified using 3'UTR sequencing. These haplotypes can be utilized for the development of CAPS markers to resolve allelic differences in 301 sites on the sweet cherry genome. These genomic resources represent a large advance in sweet cherry genomics. Potential applications of these SNPs may involve high-throughput amplicon sequencing with these primer sets using next generation sequencing technologies to obtain digital or sequence-based information in genetics studies. This is in contrast to the SNP-arrays that produce an analog signal in genotyping experiments and represent mostly intergenic polymorphisms derived from a few individuals limiting its potential applicability beyond the included polymorphisms. This methodology is expected to be of great utility in polyploid species where allele-specific haplotypes can be highly informative.

As sequencing costs plummet, the general approach reported here could be broadly implemented in identifying gene-linked polymorphisms amongst parental individuals which can then be rapidly utilized in segregation studies of a desirable set of phenotypes in the derived progeny. Polymorphisms that co-segregate with the phenotype are expected to represent the gene or set of genes that regulate the said phenotype. Establishment of these correlations is expected to open avenues for directly linking genetic and functional genomics approaches with phenomics, an emerging discipline focused on understanding genotype-phenotype relationships.

## Competing interests

The authors declare that they have no competing interests.

## Authors' contributions

TK, SS, NO, MW and AD designed the study. TK and SS prepared samples for 3'UTR sequencing. TK and VK performed assemblies and computational analysis. DJ guided and designed primers. TK analyzed the HRM curves. AH aided the computational analyses and managed data upload to NCBI. MW aided in designing primary sample collection. NO provided sweet cherry parental cultivars, segregating progeny, and guided HRM analysis. AD supervised the research and guided data interpretation. TK and AD wrote the paper. All authors read and approved the final manuscript.

## Supplementary Material

Additional file 1 **Adaptor sequences for 3'UTR sequencing**. Sequences of adaptors used in the 3'UTR sequencing of cDNA. AMID-B is an oligo-dT primer with a biotinylated 5'end. Adaptors AMID-1A to AMID12-B represent complementary oligonucleotide pairs with embedded barcode sequences. Column A is the primer name and B is the sequence. Click here for file

Additional file 2 **sup2.pl**. Custom script used to remove index sequences and rename the header with the appropriate sequence. USAGE: sup2.pl {Reads FASTA format file} {Primers/MIDs/Barcodes with corresponding headers in csv format} {# bases from start of primer to the beginning of the barcode} {New FASTA filename to be written into} Example: Input (fasta file): >1300_8769_5430 length = 258 urnand = JHSK987KJSH2KJHJK8777 AGTCCCCCGGGGTTTAAAGGGGCCCCTTTTAAAAAAGTCGTCAATGCGGT AGTCTGCAAAAAAATTTCCCCCCCCCCGGGGGGGGGGGTAGCCGTATGCA Input (MIDs csv file): Sample1,ATAGTGA Sample2,ATGCATG Output: A fasta file of the remaining sequence after removing the primer/bar code/MID with corresponding header attached as specified in the input "MIDs csv" file. Click here for file

Additional file 3 **Primers and HRM analysis**. The table represents contig number (column B), predicted amplicon length (column C), number of SNPs (column D), forward and reverse primers for each set (column E and F) used for HRM analysis. Included in the table is the Cultivar number of curve profiles (column G), number of Cowiche × Selah curve types (column H) and the Seedling number of curve profiles (column I). Click here for file

Additional file 4 **Contig sequences**. A fasta file containing the 34,620 contigs from NGen v3.0. Click here for file

Additional file 5 **Filtered SNP report**. This table is modified output generated from NGen v3.0 and SeqMan. The contig number and all details about the SNP are given including number of calls for each base at the given position from Columns B-L. Column M is the 5' flanking sequence. Column N is the polymorphism. Column O is the 3' flanking sequence. Columns M and O have been provided to enable rapid analysis of other germplasm. Click here for file

Additional file 6 **Haplotypes identified in sweet cherry**. The table presents different haplotypes identified in each contig. Some contigs have multiple positions indicated as A, B or C positions. Nucleotides corresponding to a given position in an allele are presented. Cells are merged when the differences between alleles are no longer traceable. A questions mark (?) symbolizes incomplete depth for a confirmed call at this base. Click here for file
